# Testosterone Administration Reduces Lying in Men

**DOI:** 10.1371/journal.pone.0046774

**Published:** 2012-10-10

**Authors:** Matthias Wibral, Thomas Dohmen, Dietrich Klingmüller, Bernd Weber, Armin Falk

**Affiliations:** 1 Department of Economics, University of Bonn, Bonn, Germany; 2 Center for Economics and Neuroscience, University of Bonn, Bonn, Germany; 3 Research Center for Education and the Labor Market, Maastricht University, Maastricht, The Netherlands; 4 Department of Internal Medicine I, University of Bonn, Bonn, Germany; 5 Department of Epileptology & Department of NeuroCognition, Life&Brain Center, University of Bonn, Bonn, Germany; George Mason University/Krasnow Institute for Advanced Study, United States of America

## Abstract

Lying is a pervasive phenomenon with important social and economic implications. However, despite substantial interest in the prevalence and determinants of lying, little is known about its biological foundations. Here we study a potential hormonal influence, focusing on the steroid hormone testosterone, which has been shown to play an important role in social behavior. In a double-blind placebo-controlled study, 91 healthy men (24.32±2.73 years) received a transdermal administration of 50 mg of testosterone (n = 46) or a placebo (n = 45). Subsequently, subjects participated in a simple task, in which their payoff depended on the self-reported outcome of a die-roll. Subjects could increase their payoff by lying without fear of being caught. Our results show that testosterone administration substantially decreases lying in men. Self-serving lying occurred in both groups, however, reported payoffs were significantly lower in the testosterone group (p<0.01). Our results contribute to the recent debate on the effect of testosterone on prosocial behavior and its underlying channels.

## Introduction

Telling the truth is an almost universal social norm. Likewise, lies are condemned in most societies as reflected, for example, in the eighth commandment of the Christian Decalogue and similar prescriptions in the other world religions. Proven or suspected violations of the norm to tell the truth, in particular in the form of self-serving lies, are widely considered antisocial and convey severe damages to the reputation of their author. An effective ban on lying has obvious advantages in terms of facilitating communication and the formation of trust, which is a prerequisite of economic and social exchange. Given its universal importance it is not surprising that there has been substantial interest in the prevalence and determinants of lying [1]–[3]. Despite these efforts, however, little is known about the biological foundations of lying. Here we study a potential hormonal influence, focusing on the steroid hormone testosterone.

Testosterone is known to influence brain development and reproductive physiology but also plays an important role in social behavior [4]–[9]. While most studies have investigated a potential association between testosterone and aggressive behavior, two recent studies suggest that testosterone may also increase prosocial behavior or lead to less selfish behavior in certain situations [6], [9]. We therefore investigate a link between testosterone and self-serving lying. A prominent interpretation of the existing evidence on the role of testosterone in social behavior is that the hormone enhances dominance behavior, i.e., behavior intended to gain high social status [6]–[8], [10]–[14], which in humans can be aggressive or prosocial depending on the context. Recent research suggests that pride may have evolved as an affective mechanism for motivating such status seeking behavior [15]. Pride is indirectly linked to status seeking because it is an inward directed emotion that signals high status or ego. It has been speculated that testosterone helps translate such motivation into action, for example, in acts of heroic altruism [16], [17]. Importantly, an effect of testosterone on behavior via pride should also work if behavior cannot be observed by others and an individual’s status in the eyes of the others may therefore not be directly affected.

This is precisely the case in our setup, in which subjects cannot be caught lying. As a vehicle to study lying behavior we implemented an adaptation of a simple die-rolling paradigm, which creates material incentives to lie [18]. In this task subjects are asked to roll a six-sided die once in private and to enter the result into a computer. The payoff from this task depends only on the self-reported outcome of the die roll. Subjects earn the number entered in Euro for numbers between 1 and 5, and 0 Euro for entering a 6 (see *Materials and Methods* for details). Subjects can therefore increase their payoff by lying, i.e., by reporting a higher number than they actually rolled. Importantly, the experimenter only observes the self-reported outcome and cannot tell whether an individual is actually lying.

This feature avoids problems associated with other ways of measuring lying. Asking subjects whether they lied may lead to an underreporting of lies, in particular self-serving ones, because these are viewed as socially undesirable. A setup in which lying can be directly observed may influence results for the same reason. Finally, studying lies discovered ex post can lead to biased results because the treatment intervention may not only affect the incidence of lying but also the ability to conceal a lie. In our setup, lying cannot be detected at the individual level, but it can be detected at the group level by comparing reported outcomes for each group to a uniform distribution, which is the expected distribution in the absence of lying. The paradigm is therefore well suited for a placebo-controlled study.

Most of the evidence on the effects of testosterone on human social behavior is based on correlations, which precludes causal inference. To study the causal effect of testosterone on lying behavior a total of 91 healthy young men participated in a double-blind, placebo-controlled version of the die-rolling experiment. The study lasted two days. On day 1, a single dose of 50 mg of Testogel® or a placebo gel was applied transdermally. The testosterone was allowed to load for 21–24 hours prior to the decision tasks. On day 2, subjects participated in the die-rolling task and other experiments. They were seated in separate cubicles closed off with curtains and made their decisions via a computer to ensure anonymity. After the experiment subjects filled out a questionnaire, a blood sample was taken from each subject in a separate room, and they received their payment in cash.

## Results

Testosterone administration has been successfully used to increase testosterone levels in behavioral studies with both healthy men and women [19], [20] but less is known about the pharmacokinetics of single-dose testosterone administration and potential behavioral effects in men [21] than in women [20]. We therefore conducted a manipulation check to confirm that our testosterone manipulation was effective. Plasma testosterone levels differed significantly between the testosterone and the placebo group (Mann-Whitney-U-test, p = 0.034, n = 91). In the treatment group (n = 46) the mean level of testosterone was 7.78±2.07(s.d.) ng/ml, while in the control group (n = 45) the mean level was 6.79±2.04(s.d.) ng/ml.

**Figure 1:**
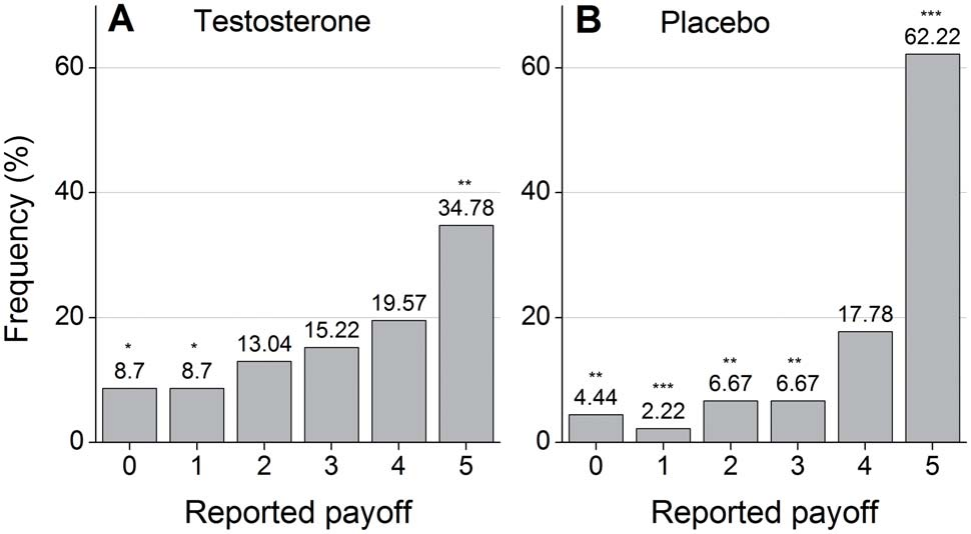
Distribution of reported payoffs. a. Distribution for subjects who received testosterone. b. Distribution for subjects who received placebo. The stars on top of the bars indicate that in the testosterone group 5s are reported significantly more frequently and 0s and 1s are reported significantly less frequently than expected. Likewise in the placebo group 5s are reported more often and 0s and 1s, 2s, and 3s are reported less frequently than expected (Binominal tests that the observed frequencies are smaller/larger than 16.7 percent, *, **, *** indicate significance at 1%, 5%, and 10% level respectively, Placebo: n = 45; Testosterone n = 46).

We observe that subjects who received testosterone report lower numbers and therefore received lower payoffs than subjects in the control condition. Figure 1 depicts the frequency of the numbers reported in the two treatments. For ease of exposition we depict reported payoffs in the graph, such that a reported die-roll of 6 is coded as a 0. Lying is prevalent in both groups as can be inferred from the fact that distributions in both groups are skewed to the right and significantly differ from a uniform distribution (Chi-square-test against uniform distribution, Placebo: p<0.001, n = 45; Testosterone: p = 0.021, n = 46). On top of the general prevalence of lying there is a strong treatment difference. The average payoff reported in the placebo group is 4.18, while it is 3.33 for the subjects with testosterone. This difference is significant (Mann-Whitney-U-test, p = 0.005, n = 91). The distributions of reported numbers differ between the testosterone and the placebo group. The treatment difference is most striking for the number 5, which is associated with the strongest material incentive to lie (62.2% in placebo vs. 34.8% in testosterone group). We test whether a payoff of 5 is reported more often in the placebo than in the testosterone group by creating a binary distribution (5 reported vs. no 5 reported) and running a Fisher-Exact test (p = 0.012, n = 91).

OLS regressions show that the effect of testosterone is robust to simultaneously including a set of potentially relevant explanatory variables such as personality variables (Big5, Machiavelli) and economic preferences (risk, delay discounting, positive and negative reciprocity, see Appendix S1 for details).

We also analyzed whether there is an overall association between testosterone levels and reported numbers. It turns out that higher testosterone levels were in fact associated with lower reported payoffs in the pooled sample (Pearson’s r = –0.22, p = 0.035, n = 91). Thus we do not only observe a treatment effect but also a significant overall correlation between testosterone level and lying.

In the questionnaire, we asked a subsample of subjects whether they thought they had received testosterone or a placebo. There was no correlation between actual and perceived testosterone administration (Pearson’s chi-square test, p = 0.94, n = 51). In addition, subjects who believed that they had received testosterone did not report significantly higher payoffs than those who believed that they had received a placebo (Mann-Whitney-U-test, p = 0.179, n = 51).

## Discussion

Our main finding is a lower incidence of self-serving lies in the testosterone group. We observe this result in a setup where subjects cannot be caught lying. To the best of our knowledge this is the first piece of evidence on a causal relationship between testosterone administration and prosocial behavior when actions are not observable to others.

Our findings contribute to the recent debate on a potential effect of testosterone on prosocial behavior [6], [9], [22]. So far, two studies suggest an effect of testosterone administration on prosocial behavior [6], [9]. However, these data are open to several interpretations regarding the underlying channel. Three hypotheses emerge from the debate [22]: 1) Testosterone has a direct influence on prosocial preferences, i.e., testosterone administration makes people more prosocial. 2) Testosterone increases concerns for social status, which may then lead to more prosocial behavior. 3) Testosterone affects beliefs about the behavior or beliefs of other players.

One study [6] finds that single-dose administration of testosterone increases proposer offers in the ultimatum game in a sample of 60 women (but see [19], [23]). The authors interpret higher offers as an expression of testosterone enhanced status concerns, i.e., proposers want to avoid status threatening rejections and therefore behave more prosocially. However, high offers cannot be interpreted as prosocial behavior per se, but may be strategically motivated. Offers in the ultimatum game depend on beliefs about the rejection behavior of the other player. Recent research suggests that testosterone could render an individual’s belief about other people more pessimistic. For example, testosterone administration reduces trustworthiness judgments of unfamiliar faces [10] and testosterone is positively related to vigilant responses to angry faces [24]. In fact, testosterone could therefore lead proposers to expect a higher rejection threshold, which would also explain higher offers in the ultimatum game. The second study opts for a different behavioral paradigm to investigate the relation between testosterone and prosocial behavior [6]. This study finds that testosterone administration increases contributions in a public good game for high 2D:4D ratio individuals in a sample of 24 women. It is possible that testosterone administration rendered subjects more prosocial. However, as in [6] it cannot be ruled out that an effect of testosterone on beliefs drives the results since the incentive structure of the public good game was such that even a perfectly selfish player with certain beliefs would contribute to the public good.

A recent summary of the debate [22] therefore concludes that “studies that are able to distinguish between the three hypotheses […] are likely to move the field forward further.” Our main contribution to this debate is therefore that, in contrast to [6] and [9], we can rule out that an effect of testosterone on beliefs is responsible for more prosocial behavior in our individual decision making paradigm. In addition, our study is the first to find an effect of testosterone on prosocial behavior in a male sample.

While we can rule out a belief effect we cannot ultimately conclude whether our findings are driven by a direct influence of testosterone on prosocial preferences or via increased status concerns. A potential interpretation for our findings is that testosterone administration affects a concern for self-image [25], or pride [16], i.e., enhances behavior which will make a subject feel proud and leads to the avoidance of behavior considered “cheap” or dishonorable. Subjects in our testosterone group may therefore lie less. This is intriguing because pride could be an affective mechanism underlying a link between testosterone and dominance behavior. An interpretation of our findings in terms of pride is in line with anecdotal and correlational evidence indicating that testosterone plays a positive part in heroic altruism [17]. It is also in line with reports that high testosterone individuals display more disobedient behavior in prison environments where proud individuals may be less willing to follow the strict rules and comply with orders [26], [27]. Finally, a relation between pride, testosterone, and the willingness to engage in “cheap” behavior also fits the observation that the five inmates with the lowest testosterone levels in a sample of 87 female prison inmates were characterized as “sneaky” and “treacherous” by prison staff members [27]. Further experiments manipulating whether lying is an honorable action (e.g., lying for charity) or not (lying for self) are needed to clarify the role of pride in the effect of testosterone on human social behavior. An alternative interpretation of our results, which we cannot rule out, is that testosterone has a direct effect on prosocial behavior, making people more honest per se.

At the current stage, we can only speculate about the neural mechanisms underlying the effect of testosterone administration on lying. A recent study suggests that testosterone may affect behavior via reduced activity in the orbitofrontal cortex (OFC) [28]. Evidence from neuroimaging and lesion studies suggests that the prefrontal cortex also plays an important role in lying [29]. For example, the OFC and the anterior cingulate cortex were significantly activated during deception in healthy young men [30]. The effect of testosterone on lying in the present study may therefore be the result of reduced OFC activity.

A potential caveat is that our treatment effect may be a “placebo effect”. If subjects in one group are more likely to think that they have received testosterone and have a strong belief about the effect of testosterone a treatment difference could be observed even in the absence of a pure substance effect [6]. However, there was no correlation between perceived and actual testosterone administration. We also find no evidence that beliefs about testosterone administration influenced behavior.

We deliberately chose a design in which subjects cannot get caught lying (see above). A second potential limitation of our study is therefore that we cannot rule out with certainty the possibility that our subjects were telling the truth. While this is statistically implausible, future studies should nevertheless complement ours with a different paradigm in which it is possible to detect lying at the individual level.

## Materials and Methods

### Subjects

The study was approved by the ethics committee of the University Hospital of the University of Bonn. 91 healthy men (age 24.32±2.73(s.d.) years) gave written informed consent prior to inclusion into the study. All subjects were screened to exclude benign prostate hypertrophy, prostate cancer, heart failure, renal failure, hepatic failure, epilepsy or migraine history, and exogenous uptake of cortisone or ACTH. No adverse events occurred.

### Experimental Procedure

The study used a double-blind, placebo-controlled design. There was no deception in any part of this experiment. Each session of the study took place on two consecutive days. On day 1, subjects reported individually to the Institute for Empirical Research in Economics at the University of Bonn between 10 am and 1 pm. After receiving general instructions subjects were randomly assigned to the testosterone or the placebo group and 50 mg of Testogel® or a placebo gel were applied on their upper right arm. Afterwards participants had to wait at the Institute until the gel was fully absorbed (approx. 10 min) before leaving the Institute. Subjects were instructed to refrain from showering or swimming for at least 6 hours after the transdermal application, to avoid drinking alcohol until the end of the experiment and to obtain enough sleep. The testosterone was allowed to load for 21–24 hours prior to the decision tasks. On day 2, subjects reported to the BonnEconLab at 10 am to participate in several unrelated experiments. Subjects were seated in separate cubicles closed off with curtains and read self-paced instructions for the experiments (available from the authors upon request). Questions were rare and answered in private. All experiments were administered using ztree software [31].

Before the lying experiment [18] the experimenter distributed a six-sided die to every subject. Subjects were encouraged to roll the die several times before the experiment started to check that it had not been manipulated. Instructions for the experiment were given on screen. The instructions stated explicitly that numbers entered would not be controlled by the experimenter. Subjects were asked to roll the die once when prompted and to enter their result into a mask on screen. The payoff was the number entered in Euro for numbers between 1 and 5, and 0 for a 6.

After the experiments subjects answered a questionnaire on socio-demographic and personality characteristics. Finally, a blood sample was taken from each subject in a separate room and subjects received the payoff from all experiments plus a base fee of 40 Euro in cash. The sessions on day 2 lasted on average 150 minutes including blood sampling and payment procedures. Five sessions were conducted in total. Neither the research assistant on day 1 nor the experimenter on day 2 knew which subjects in a session belonged to the testosterone or the placebo group respectively.

### Testosterone Measurement

The blood samples were stored at the hormone laboratory of the gynecology department at the University of Bonn and processed within a day after collection for measurement of total testosterone using a one-step Chemieluminescent Micropparticle Immunoassay (ARCHITECT Testosterone, Abbott Laboratories, Wiesbaden, Germany [32]). The intraassay coefficient and the interassay coefficients were 1.9% and 3.7% respectively, with a lower detection limit of 0.14 ng/ml.

Testosterone administration resulted in a significant difference in plasma testosterone levels (Mann-Whitney-U-test, p = 0.034, n = 91). In the treatment group (n = 46) the mean level of testosterone was 7.78±2.07(s.d.) ng/ml, while the control group (n = 45) had a mean level of 6.79±2.04(s.d.) ng/ml. These figures likely underestimate the true difference in testosterone levels at the time of the die-rolling experiment given that blood samples were taken about 45 min later, at the end of the session. In addition, based on [32] the maximum difference in testosterone serum levels is likely to have occurred earlier.

### Questionnaires

After the experiment, subjects answered several questionnaires. We use a validated short German version of the Big Five personality inventory [33] and the Mach IV scale [34] to assess subjects’ personality traits. To measure economic preferences regarding risk-taking, we included an experimentally validated question on general willingness to take risks [35]. Two questions taken from the German Socioeconomic Panel were used to assess self-reported impatience and impulsivity. In addition, subjects answered a six item questionnaire on positive and negative reciprocity [36]. For a subset of subjects we also elicited at the end of the questionnaire whether they believed they had received testosterone or placebo. All subjects completed a questionnaire on socio-demographic characteristics.

### Statistical Analysis

Our statistical analysis is based on non-parametric Mann-Whitney U-tests, binomial tests, chi-square tests and Fisher exact tests, and parametric t-tests. All tests are two-tailed tests. OLS regressions are used for additional robustness checks (see table S1).

## Supporting Information

Table S1 **OLS regressions of reported payoff on an indicator variable for testosterone administration, age,and measures of economic preferences and different personality variables.**
(DOC)

Appendix S1 **Supporting statistics and results.**
(DOC)
