# Supplementary material for: Testosterone Administration Reduces Lying in Men
Source: PLoS One. 2012 Oct 10;7(10):e46774. doi: 10.1371/journal.pone.0046774 (PMC3468628; doi:10.1371/journal.pone.0046774)
Supplement: Table S1 — OLS regressions of reported payoff on an indicator variable for testosterone administration, age,and measures of economic preferences and different personality variables. (DOC) [file pone.0046774.s001.doc]

**Table S1. OLS regressions of reported payoff on an indicator variable for testosterone administration (1) and age (2) and measures of economic preferences (3) and different personality variables (4).**

|  | **(1)** | **(2)** | **(3)** | **(4)** |
| --- | --- | --- | --- | --- |
| Testosterone | -.852***  (.321) | -.851***  (.320) | -.788**  (.326) | -.828***  (.311) |
| Age |  | -.074  (.059) | -.084  (.059) | -.086  (.061) |
| General willingness to take risks |  |  | .080  (.087) | .072  (.085) |
| Impatience |  |  | -.019  (.068) | .016  (.066) |
| Impulsivity |  |  | .141*  (.075) | .194**  (.083) |
| Positive reciprocity |  |  | -.107  (.079) | -.070  (.078) |
| Negative Reciprocity |  |  | .058  (.038) | .053  (.039) |
| Conscientiousness |  |  |  | -.031  (.053) |
| Extraversion |  |  |  | -.098**  (.047) |
| Agreeableness |  |  |  | -.018  (.078) |
| Openness |  |  |  | -.078  (.049) |
| Neuroticism |  |  |  | -.086  (.044) |
| Machiavelli |  |  |  | .026  (.019) |
| Constant | 4.178***  (.228) | 5.970***  (1.452) | 6.504***  (2.325) | 8.261 **  (3.398) |
| Adjusted R^2^ | 0.0629 | 0.0687 | 0.1197 | 0.2036 |
| N | 91 | 91 | 91 | 91 |

*** and ** denote significance at the 1% and 5% level respectively. Standard errors are given in parenthesis.
