# Supplementary material for: Testosterone Administration Reduces Lying in Men
Source: PLoS One. 2012 Oct 10;7(10):e46774. doi: 10.1371/journal.pone.0046774 (PMC3468628; doi:10.1371/journal.pone.0046774)
Supplement: Appendix S1 — Supporting statistics and results. (DOC) [file pone.0046774.s002.doc]

**Appendix S1 - Supporting Statistics and Results**

*Controlling for economic preferences and personality variables*

In the post-experimental questionnaire we elicited several measures of economic preferences previously used in the German Socioeconomic Panel including subjects’ general willingness to take risks, impatience, impulsivity and positive and negative reciprocity. The questions regarding risk, impatience, and impulsivity were answered on a scale from 0 (not at all willing to take risks/impatient/impulsive) to 10 (very willing to take risks/very impatient/ very impulsive). Positive and negative reciprocity were assessed with a 6-item questionnaire (3 items each) in which subjects had to state their agreement with a sentence on a scale from 1 to 7 (e.g. “If somebody puts me in a difficult situation, I will do the same to her/him.”). Scores for positive and negative reciprocity were obtained by adding answers for the individual items. We conduct a robustness check of our main result by regressing reported payoff on an indicator variable equal to 1 if a subject received testosterone, age, and our preference and personality measures (see Table S1). Note that the coefficient of the indicator variable stays significant and changes only slightly between the different specifications. Thus, we observe significant differences in the reported payoffs between the placebo and the testosterone group, even controlling for age, preference and personality measures.

**Table S1. OLS regressions of reported payoff on an indicator variable for testosterone administration (1) and age (2) and measures of economic preferences (3) and different personality variables (4).**

|  | **(1)** | **(2)** | **(3)** | **(4)** |
| --- | --- | --- | --- | --- |
| Testosterone | -.852***  (.321) | -.851***  (.320) | -.788**  (.326) | -.828***  (.311) |
| Age |  | -.074  (.059) | -.084  (.059) | -.086  (.061) |
| General willingness to take risks |  |  | .080  (.087) | .072  (.085) |
| Impatience |  |  | -.019  (.068) | .016  (.066) |
| Impulsivity |  |  | .141*  (.075) | .194**  (.083) |
| Positive reciprocity |  |  | -.107  (.079) | -.070  (.078) |
| Negative Reciprocity |  |  | .058  (.038) | .053  (.039) |
| Conscientiousness |  |  |  | -.031  (.053) |
| Extraversion |  |  |  | -.098**  (.047) |
| Agreeableness |  |  |  | -.018  (.078) |
| Openness |  |  |  | -.078  (.049) |
| Neuroticism |  |  |  | -.086  (.044) |
| Machiavelli |  |  |  | .026  (.019) |
| Constant | 4.178***  (.228) | 5.970***  (1.452) | 6.504***  (2.325) | 8.261 **  (3.398) |
| Adjusted R^2^ | 0.0629 | 0.0687 | 0.1197 | 0.2036 |
| N | 91 | 91 | 91 | 91 |

*** and ** denote significance at the 1% and 5% level respectively. Standard errors are given in parenthesis.
